# Mitochondrial Transfer of Wharton's Jelly Mesenchymal Stem Cells Eliminates Mutation Burden and Rescues Mitochondrial Bioenergetics in Rotenone-Stressed MELAS Fibroblasts

**DOI:** 10.1155/2019/9537504

**Published:** 2019-05-22

**Authors:** Tsu-Kung Lin, Shang-Der Chen, Yao-Chung Chuang, Min-Yu Lan, Jiin-Haur Chuang, Pei-Wen Wang, Te-Yao Hsu, Feng-Sheng Wang, Meng-Han Tsai, Sheng-Teng Huang, Xiao-Wen Wang, Po-Chin Tsai, Hung-Yu Lin, Chia-Wei Liou

**Affiliations:** ^1^Department of Neurology, Kaohsiung Chang Gung Memorial Hospital and Chang Gung University College of Medicine, Kaohsiung 833, Taiwan; ^2^Mitochondrial Research Unit, Kaohsiung Chang Gung Memorial Hospital and Chang Gung University College of Medicine, Kaohsiung 833, Taiwan; ^3^Center of Parkinson's Disease, Kaohsiung Chang Gung Memorial Hospital and Chang Gung University College of Medicine, Kaohsiung 833, Taiwan; ^4^Department of Pediatric Surgery, Kaohsiung Chang Gung Memorial Hospital and Chang Gung University College of Medicine, Kaohsiung 833, Taiwan; ^5^Department of Internal Medicine, Kaohsiung Chang Gung Memorial Hospital and Chang Gung University College of Medicine, Kaohsiung 833, Taiwan; ^6^Department of Obstetrics and Gynecology, Kaohsiung Chang Gung Memorial Hospital and Chang Gung University College of Medicine, Kaohsiung 833, Taiwan; ^7^Core Laboratory for Phenomics and Diagnostic, Chang Gung University College of Medicine, Kaohsiung, Taiwan; ^8^Department of Medical Research, Chang Gung University College of Medicine, Kaohsiung, Taiwan; ^9^Department of Chinese Medicine, China Medical University Hospital, Taichung, Taiwan

## Abstract

Wharton's jelly mesenchymal stem cells (WJMSCs) transfer healthy mitochondria to cells harboring a mitochondrial DNA (mtDNA) defect. Mitochondrial myopathy, encephalomyopathy, lactic acidosis, and stroke-like episodes (MELAS) is one of the major subgroups of mitochondrial diseases, caused by the mt.3243A>G point mutation in the mitochondrial tRNALeu^(UUR)^ gene. The specific aim of the study is to investigate whether WJMSCs exert therapeutic effect for mitochondrial dysfunction in cells of MELAS patient through donating healthy mitochondria. We herein demonstrate that WJMSCs transfer healthy mitochondria into rotenone-stressed fibroblasts of a MELAS patient, thereby eliminating mutation burden and rescuing mitochondrial functions. In the coculture system *in vitro* study, WJMSCs transferred healthy mitochondria to rotenone-stressed MELAS fibroblasts. By inhibiting actin polymerization to block tunneling nanotubes (TNTs), the WJMSC-conducted mitochondrial transfer was abrogated. After mitochondrial transfer, the mt.3243A>G mutation burden of MELAS fibroblasts was reduced to an undetectable level, with long-term retention. Sequencing results confirmed that the transferred mitochondria were donated from WJMSCs. Furthermore, mitochondrial transfer of WJMSCs to MELAS fibroblasts improves mitochondrial functions and cellular performance, including protein translation of respiratory complexes, ROS overexpression, mitochondrial membrane potential, mitochondrial morphology and bioenergetics, cell proliferation, mitochondrion-dependent viability, and apoptotic resistance. This study demonstrates that WJMSCs exert bioenergetic therapeutic effects through mitochondrial transfer. This finding paves the way for the development of innovative treatments for MELAS and other mitochondrial diseases.

## 1. Introduction

Mitochondria are organelles responsible for the production of ATP, the major energy currency of the cell. In humans, mitochondrial dysfunction results in metabolic imbalance, intracellular ATP deficiency, reactive oxygen species (ROS) production, and perturbation in cell death singling [1, 2]. Mitochondrial DNA (mtDNA) is an approximately 16.6 kilobase, double-stranded, circular molecule encoding 37 genes, with several thousand copies per cell in humans [3]. Mutations in mtDNA may cause a broad spectrum of multisystemic diseases. Many patients of mitochondrial diseases harbor both normal and mutant mtDNA in a single cell, a state known as heteroplasmy. The degree of heteroplasmy and distribution of mutant mtDNA in the patient's tissues determine the severity and phenotypic heterogeneity of the disease [4].

Mitochondrial myopathy, encephalomyopathy, lactic acidosis, and stroke-like episodes (MELAS) is one of the major clinical subgroups of such mitochondrial diseases, caused by point mutations: mt.3243A>G, mt.3271T>C, mt.13513G>A, and others [5]. The mt.3243A>G mutation at mt-tRNALeu^(UUR)^ in particular has been associated with certain defects, including impaired transcription termination [6], decreased half-life of tRNALeu^(UUR)^ molecules [7], and abnormal tRNA folding [8]. These defects could negatively influence mitochondrial translation and consequently hamper oxidative phosphorylation (OXPHOS) and bioenergetics in MELAS cells. Typical manifestations of MELAS syndrome include stroke-like episodes, seizures, dementia, diabetes, ataxia, epilepsy, optic atrophy, deafness, migraine, cortical blindness, cardiomyopathy, myopathy, exercise intolerance, lactic acidosis, and vomiting [9]. Cells from MELAS patient harboring the mt.3243A>G mutation have been shown to present markedly decreased activity of respiratory chain (RC) complexes [10–12] and increased activity of antioxidant enzymes, superoxide dismutase, and catalase [13]. The deficient RC complexes may contribute to inefficient ETC and ultimately ROS leak. Accordingly, the increased activity of antioxidant enzymes could be regarded as a compensatory response to elevated ROS production.

There is an increasing interest in the therapeutic potential of mesenchymal stem cells (MSCs) in treating mitochondrial disorder. Spees et al. first demonstrated that bone marrow-derived MSCs (BMMSCs) perform mitochondrial transfer to replenish mtDNA-devoid *ρ* ^0^ pulmonary adenocarcinoma cells with mtDNA to rescue mitochondrial functions [14]. Subsequently, BMMSCs have been reported to exert therapeutic effects in acute lung injury through intercellular mitochondrial transfer [15]. It has been shown that tunneling nanotubes (TNTs) serve as channels transporting intracellular components between connected cells both *in vitro* and *in vivo* [16]. These components range from cytoplasm, ions, lipid droplet, viral and bacterial pathogens, and organelles such as mitochondria and lysosomes [17, 18].

Although BMMSCs are the most common source of therapeutic MSCs, umbilical cord-derived Wharton's jelly MSCs (WJMSCs) provide an alternative, with more accessibility and fewer ethical constraints than BMMSCs. Furthermore, WJMSCs present a rapid proliferation rate, notable expansion capability, no tumorigenicity, and strong immunomodulatory capacities [19, 20]. Our team previously reported that umbilical cord-derived WJMSCs successfully transfer mitochondria into *ρ* ^0^ osteosarcoma cells [21], as well as cybrid cells harboring the mt.8344A>G mutation of a myoclonic epilepsy with ragged-red fibers (MERRF) patient [22]. Mitochondrial transfer from WJMSCs to MERRF cybrid cells ameliorated mutation burden, oxidative stress, and enhanced bioenergetics [22].

In this study, we aimed to investigate whether WJMSC-conducted mitochondrial transfer could mitigate mutation burden and rescue mitochondrial dysfunction in a MELAS patient's fibroblasts. We demonstrated that in a separate coculture, mitochondrial transfer from WJMSCs to rotenone-stressed MELAS fibroblasts eliminated mutation burden through a TNT-dependent manner, with long-term retention. Mitochondrial transfer to MELAS fibroblasts resulted in improved mitochondrial translation, oxidative stress, fusion-prone morphology, bioenergetics, cell proliferation, mitochondrion-dependent viability, and antiapoptotic resistance. This effectively provides the opportunity for correcting mitochondrial mutation burden of MELAS and other mitochondrial diseases through WJMSC-based mitochondrial therapy.

## 2. Materials and Methods

### 2.1. Fibroblast Culture

Fibroblasts were isolated from a patient skin punch biopsy. After a briefing and the signing of informed consent, the patient underwent a skin punch biopsy performed by an experienced physician using a punch gun (4 mm Biopsy Punches, Part Numbers 33-34, Integra™ Miltex®). The dissected skin biopsy pieces were quickly transferred into a sterilized bottle containing PBS (Gibco) and 2% antibiotics (Gibco). The skin biopsy pieces were then diced into smaller pieces and transferred to DMEM (Gibco) supplemented with FBS (Gibco), GlutaMAX (Gibco), and 1% antibiotics in a tissue culture plate, after which they were not disturbed. After fibroblast colonies emerged around the skin tissue, internal trypsination was performed to facilitate cell expansion. Single clones were isolated using limiting dilution. Briefly, 2,000 fibroblasts were seeded in well A1 of a 96-well plate, followed by first vertical dilution series (from A1 to well H1) using a single-channel pipette and then second horizontal dilution series (from column 1 to column 12) using an 8-channel pipette in a 1 : 2 ratio. Final medium volume of each well was kept in 200 *μ*l. After two to three weeks, single-cell clone was transferred to 12-well plate and then to 60 mm dish in a progressive manner. Expanded cells were subjected to genotyping for the mt.3243A>G mutation burden. The study protocol and informed content were reviewed and approved by the Institutional Review Board of Chang Gung Memorial Hospital (CGMH; IRB No. 104-5205C).

### 2.2. Isolation, Cultivation, and Identification of WJMSCs

Human WJMSCs were prepared from the postpartum human umbilical cords obtained during normal spontaneous deliveries after informed consent had been obtained. The preparation of human WJMSCs has previously been described [21]. Briefly, the human umbilical cords were placed in Hanks' balanced salt solution (Gibco) supplemented with 2% antibiotics (Gibco) before harvesting of the WJMSCs. After the arteries and veins had been removed, the remaining cord was diced into small pieces and transferred to 100 mm dishes containing DMEM supplemented with 10% FBS in a 37°C incubator at 5% CO_2_. Upon reaching 100% confluence, cells were detached using 0.25% trypsin-EDTA (Gibco). The WJMSCs had a typical spindle-shaped appearance and presented positive for CD73, CD90, and CD105, while negative for CD31, CD34, and CD45. FITC-conjugated antibodies, including anti-CD73 (410200, Invitrogen), anti-CD90 (#GTX11155, GeneTex), anti-CD105 (#GTX11415, GeneTex), anti-CD31 (#GTX43363, GeneTex), anti-CD34 (#GTX18227, GeneTex), and anti-CD45 (#MHCD4520, Invitrogen), were used for the detection of surface markers and analyzed in a flow cytometry (FACSCalibur, BD Biosciences). Study protocol and informed consent were reviewed and approved by the Institutional Review Board of Chang Gung Memorial Hospital (CGMH; IRB No. 101-1620A3).

### 2.3. Coculturing and Imaging

WJMSCs and MELAS fibroblasts were transfected with Cox4-DsRed and Su9-EGFP plasmids, respectively, using the Lipofectamine® 3000 Transfection Reagent (Invitrogen) prior to coculture. After 24 h of coculture, mitochondrial transfer was counted under a fluorescence microscope (Leica, Wetzlar, Germany). The role of TNTs of WJMSCs in mediating mitochondrial transfer was validated in the presence or absence of cytochalasin B (#C2743, Sigma) at 350 nM, 24 h prior to coculture with MELAS fibroblasts.

### 2.4. Measurement of mt.3243A>G Mutation Burden

Mutation burden detection was performed as previously described [23]. Briefly, total DNA of cultured cells were extracted using Quick-DNATM Miniprep kit (Zymo Research). The primers used were L2678 (2678-2696): 5′-ATTGACCTGCCCGTGAAGA-3′ and H3836 (3836-3817): 5′-GGCAGGAGTAATCAGAGGTG-3′. The amplified PCR products (1159 bp) were then subjected to restriction digestion by *Apa* I (Thermo Fisher Scientific), which can recognize the restriction site (5′-GGGCCC-3′) created by the A3243G mutation to form a 591 bp and a 568 bp fragment. The PCR products were loaded onto 0.7% agarose gel in Tris-acetate EDTA (TAE) buffer containing 0.01% of SYBR safe DNA Gen Stain (Invitrogen). After electrophoresis, the gels were photographed under ultraviolet light. The proportion of the mt.3243A>G mutation burden was quantified with ImageJ.

### 2.5. Measurement of ROS Production

The measurements of intracellular and mitochondrial ROS were determined with flow cytometry, following cell staining with CM-H_2_DCFDA (Invitrogen) and MitoSOX™ Red (Invitrogen) fluorescent probe, respectively. Cells were washed twice with PBS and stained with CM-H_2_DCFDA (5 *μ*M) or MitoSOX™ Red (5 *μ*M) for 30 min at 37°C. Cell pellets were collected, washed twice with PBS, and then resuspended in PBS. Fluorescence was detected with FACSCalibur flow cytometer (BD Biosciences).

### 2.6. Measurement of Mitochondrial Membrane Potential (MMP)

MMP was evaluated using cationic fluorescent dye tetramethylrhodamine ethyl ester (#T-3168; Thermo Scientific). Cells were incubated in 100 nM TMRE for 30 min at 37°C. They were then washed twice with 1x PBS, and fluorescence signal was examined and photographed by a fluorescence microscope. For quantitative measurement, cells stained by TMRE were analyzed using flow cytometer (FACSCalibur, BD Biosciences).

### 2.7. Western Blot

Cells were lysed in RIPA lysis buffer (#R0278, Sigma) with the addition of the Halt Protease Inhibitor Cocktail (#8778, Thermo Scientific) and Halt Phosphatase Inhibitor Cocktail and EDTA solution (#78420, Thermo Scientific). The antibodies used included anti-NDUFA9 (#20312-1-AP, Proteintech), anti-COX4 (#GTX114330, GeneTex), anti-COX2 (#55070-1-AP, Proteintech), anti-*β*-actin (#MAB1501, Millipore), peroxidase-conjugated AffiniPure Goat Anti-Mouse IgG (H+L) (#115-035-003, Jackson ImmunoResearch), and peroxidase-conjugated AffiniPure Goat Anti-Rabbit IgG (H+L) (#111-035-003, Jackson ImmunoResearch). The signals were developed using the Ultra ECL-HRP Substrate (#TU-ECL02, TOOLS) using X-ray films.

### 2.8. Detection and Categorization of Mitochondrial Morphology

The examination and quantification of mitochondrial morphology were conducted as previously described [24]. Briefly, mitochondria were visualized using mitochondrial-targeting fluorescent protein Cox4-DsRed, generously provided by Dr. David Chan (California Institute of Technology, Pasadena, CA 91125, USA). The MicroP algorithm was utilized to categorize mitochondrial morphology into fission (small globe) and fusion types (simple tube, twisted tube, donut tube, and branching tube) [25]. *N* = 75–400 mitochondria were obtained from 10–30 cells and three independent experiments.

### 2.9. ATP Assay

7.5 × 10^4^ cells were trypsinized, washed, and resuspended in DPBS (Invitrogen) supplemented with 2% FBS and incubated in the presence of DMSO or oligomycin (Sigma) at 37°C for 2 h. Cells were then collected to determine ATP level (K354-100, BioVision) according to the manufacturer's guidelines.

### 2.10. Oxygen Consumption Rate (OCR)

Oxygen consumption measurements were performed in a Seahorse XF24 Analyzer (Agilent). 2 × 10^4^ cells were seeded in each well of a Seahorse Flux Analyzer plate. Cells were incubated in DMEM in Seahorse cell plates for 1 h before oxygen consumption measurement. When assay was performed, three measurements of the basal level of oxygen consumption were recorded. Subsequently, oligomycin (1 *μ*M), a complex V inhibitor, was injected and mixed, and three measurements were recorded to determine ATP-linked oxygen consumption and proton leak. Following oligomycin, FCCP (0.25 *μ*M), a proton uncoupler, was injected and mixed, and another three measurements were recorded to determine maximal respiration capacity. Finally, complex I inhibitor rotenone (0.5 *μ*M) and complex III inhibitor antimycin A (0.5 *μ*M) were injected and mixed, and three measurements were recorded to determine nonmitochondrial oxygen consumption.

### 2.11. Cell Proliferation

For measurement of cell proliferation rate, cells were seeded in high-glucose DMEM (Gibco) and cell viability was determined by Cell Counting Kit-8 at days 0 and 7.

### 2.12. Mitochondrion-Dependent Viability Assay

1 × 10^4^ cells were seeded in a 12-well plate. Culture medium was replaced with glucose-free DMEM supplemented with 10 mM galactose (Sigma) and 10% FBS on the day prior to day 0. Bright-field cellular morphology was observed under a microscope (Leica, Wetzlar, Germany) at days 0, 5, and 7. Cell viability was determined by Cell Counting Kit-8 (Sigma).

### 2.13. Determination of Cell Apoptosis

Cells were fixed with 4% paraformaldehyde for 15 min at room temperature. After PBS washing twice, cells were permeabilized with 0.1% Triton X-100 (Sigma-Aldrich) diluted in PBS for 15 min. For detection of cleaved caspase-3, following blocking with 2% of BSA (Sigma-Aldrich) for 4 h, primary antibody against cleaved caspase-3 (Asp 175) (#9759, Cell Signaling) was added. The cells were incubated overnight at 4°C. Goat anti-rabbit secondary antibody (#R37117, Invitrogen) was added for 1 h at room temperature. The nuclei were stained with DAPI (#D1306, Invitrogen) diluted in PBS for 5 min and mounted with the ProLong™ Gold Antifade Mountant (#P36934, Invitrogen). Cleaved caspase-3-positive percentage was counted from at least three randomized fields of three independent experiments. For detection of DNA strand breaks, terminal deoxynucleotidyl transferase-mediated nick-end labeling (TUNEL) staining was conducted by an *In Situ* Cell Death Detection Kit (#11684795910, Roche) according to manufacturer's protocol. Images were acquired with a fluorescence microscope (Leica, Wetzlar, Germany). TUNEL signal intensity was quantified by ImageJ.

### 2.14. Statistical Analysis

Data collected from at least three independent experiments were expressed as the mean ± SEM. Differences between two data sets were evaluated by two-tailed unpaired Student's *t*-tests. Statistical tests between multiple data sets were analyzed using a one-way analysis of variance (ANOVA) followed by post hoc Bonferroni's test. A *p* value under 0.05 was considered statistically significant.

## 3. Results

### 3.1. MELAS Fibroblasts Exhibit Mitochondrial Dysfunction and Increased ROS Production in a Mutation Burden-Dependent Manner

**Figure 1:**
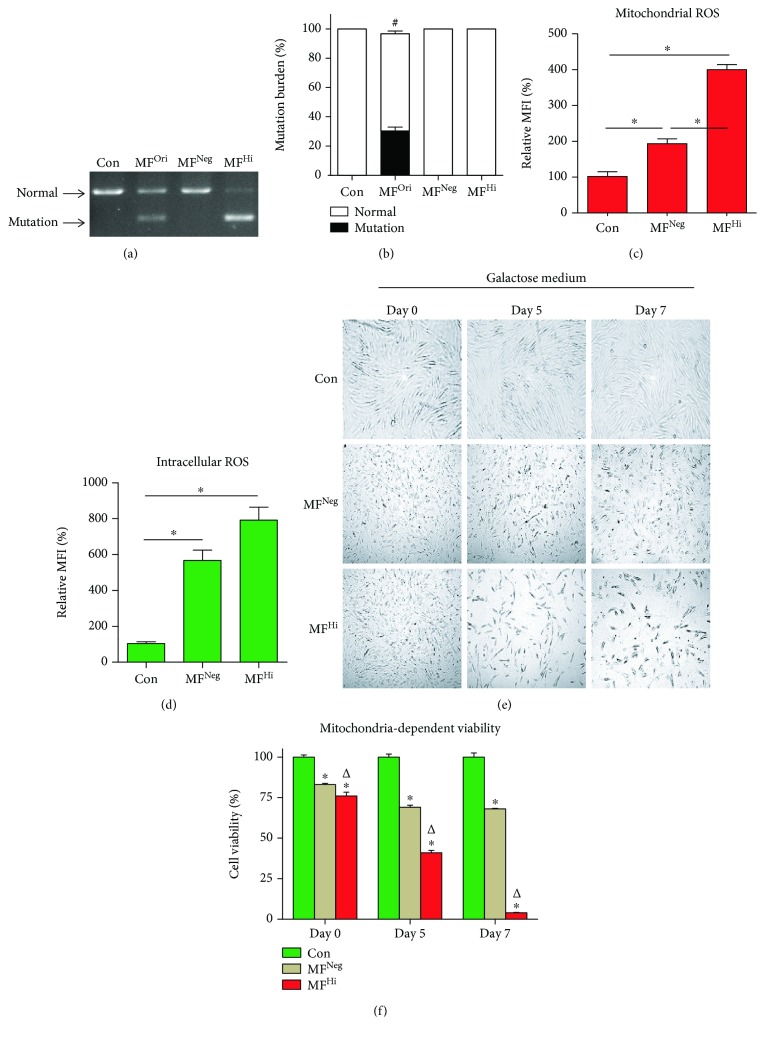
MELAS patient-derived fibroblasts exhibit mitochondrial dysfunction and increased reactive oxygen species (ROS) expression in a mutation burden-dependent manner. (a) Representative image of the mt.3243A>G mutation burden of each fibroblast clone. The mutation (mt3243A>G) was detected using PCR-RFLP. PCR product containing normal mt.3243A was not digested by *Apa I* and showed an 1159 bp band. The mt.3243A>G mutation was *Apa I*-cleaved into 598 and 591 bp band. (b) Relative proportions of the mt.3243A>G mutation burden. (c, d) Mitochondrial and intracellular ROS were detected with MitoSOX™Red and H_2_DCFDA, respectively. (e) Representative image of mitochondrion-dependent viability. Glucose-containing culture medium was replaced with glucose-free 10 mM galactose medium at day 0. Cell confluence photographed and viability detected with CCK-8 kit at days 0, 5, and 7. ^#^ *p* < 0.05 significantly different when compared to all experimental groups. ^∗^ *p* < 0.05 significantly different when compared to the indicated group. Con: control fibroblast from normal human; MF^Ori^: original MELAS fibroblast derived from patient; MF^Neg^: MELAS fibroblast clone harboring negative mutation burden; MF^Hi^: MELAS fibroblast clone harboring high mutation burden; MFI: mean fluorescence intensity.

To verify whether the mt.3243A>G mutation is the cause of mitochondrial dysfunction, we first obtained MELAS fibroblasts harboring various degrees of mutation burden through clonal isolation (Figure S1). MELAS fibroblasts with negative mutation burden (MF^Neg^) presented undetectable mutation signals, identical to healthy control fibroblasts, while fibroblasts with high mutation burden (MF^Hi^) harbored the mt.3243A>G at a rate of up to 80% (Figures 1(a) and 1(b)). Consistent with mutation burden, MF^Hi^ cells presented higher mitochondrial and total intracellular ROS production than MF^Neg^ cells (Figures 1(c) and 1(d)). Galactose medium, in which cells with normal mitochondria are able to survive [26], was then utilized to examine mitochondrion-dependent viability. In contrast to the competent viability characteristics of the control and MF^Neg^ cells, MF^Hi^ cells presented cell loss over time (Figures 1(e) and 1(f)), indicating that the high mutation burden contributes to mitochondrial dysfunction.

### 3.2. Mitochondrial Transfer from WJMSCs to MELAS Fibroblasts Pretreated with Rotenone Eliminates Mutation Burden with Long-Term Retention

**Figure 2:**
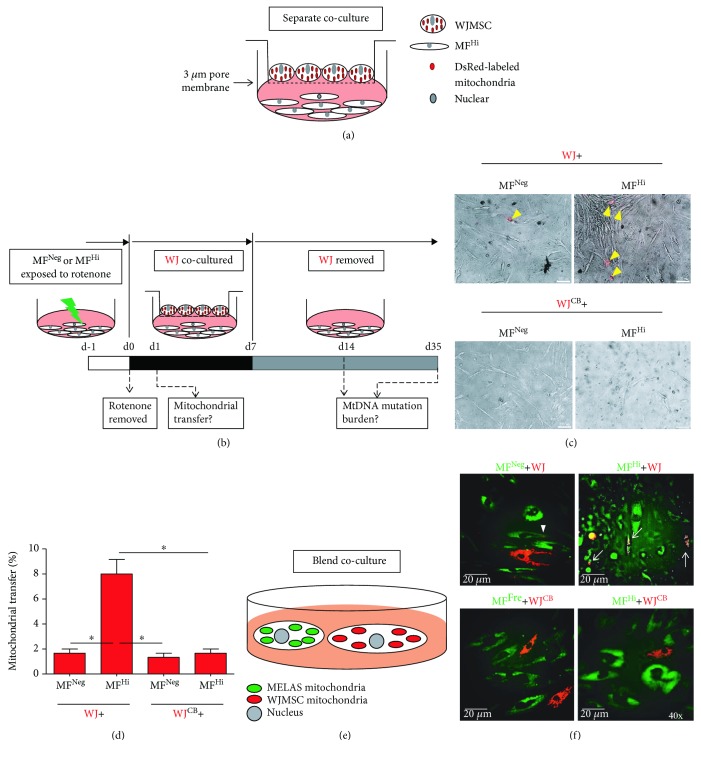
Mitochondrial transfer from WJMSCs to MELAS fibroblasts pretreated with rotenone. (a) Schematic of a separate coculture in which a 3 *μ*m pore membrane divides Cox4-DsRed-expressing WJMSCs from MELAS fibroblast. (b) Experimental course shows that MELAS fibroblasts pretreated with 500 nM rotenone for 24 h, followed by a 7-day coculture. Mitochondrial transfer and mtDNA mutation burden were examined at the indicated time point. (c) Representative image of mitochondrial transfer from WJMSCs to MELAS fibroblast. Arrowheads indicate mitochondrial transfer of WJMSCs into MELAS fibroblast. (d) Quantitative results of mitochondrial transfer efficiency. (e) Schematic of the blended coculture. WJMSCs from MELAS fibroblast, respectively, labeled with mitochondrial Cox-DsRed and Su9-EGFP were subjected to blend coculture for one day. (f) Representative image of intercellular mitochondrial transfer. Arrowheads indicate mitochondrial transfer of WJMSCs. Arrows indicate hybrid mitochondria. ^∗^ *p* < 0.05 significantly different when compared to any other group. WJ: Wharton's jelly mesenchymal stem cell; MF^Neg^: MELAS fibroblast clone harboring negative mutation burden; MF^Hi^: MELAS fibroblast clone harboring high mutation burden; WJ^CB^: WJMSCs pretreated with 350 nM cytochalasin B for 24 h before coculture.

**Figure 3:**
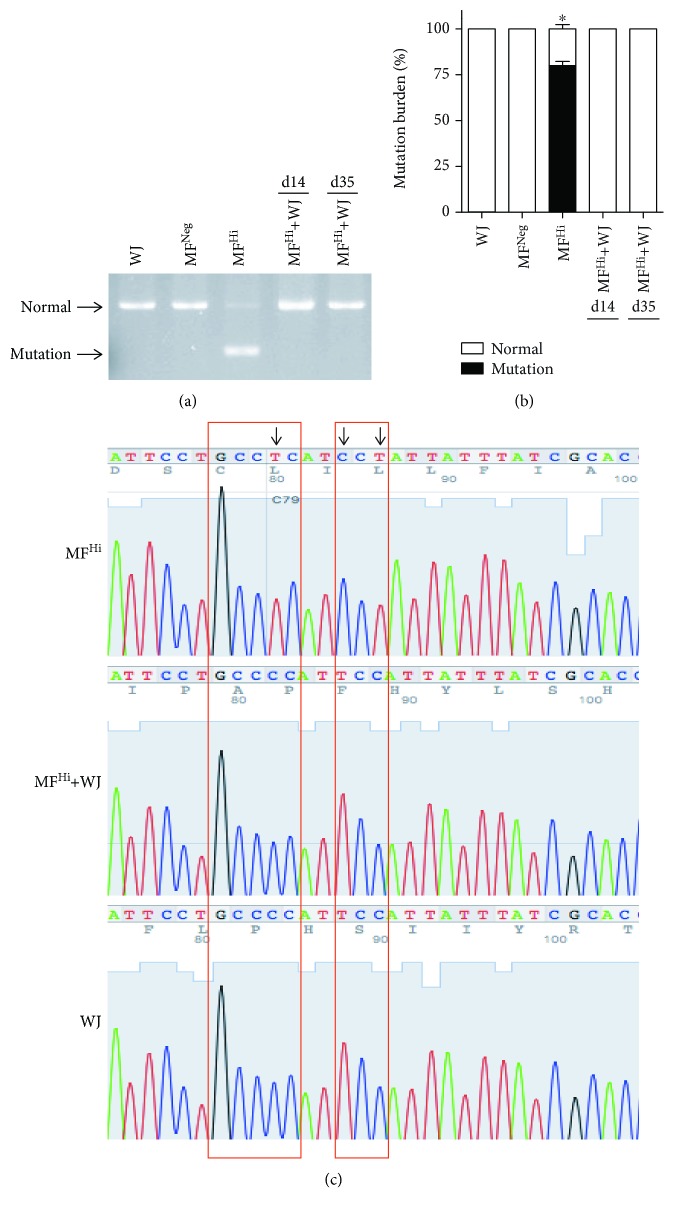
Mitochondrial transfer from WJMSCs eliminates mutation burden of MELAS fibroblasts with long-term retention. Experimental course is shown in Figure 2(b). (a) Representative result of mutation burden after a separate coculture. (b) Relative proportions of mutation burden. (c) Identification of mitochondrial genotype by sequencing HVR2 segment of mtDNA. Arrows indicate that mtDNA genotype of MF^Hi^+WJ is identical to WJ, but not to MF^Hi^. ^∗^ *p* < 0.05 significantly different when compared to all experimental groups. WJ: Wharton's jelly mesenchymal stem cell; MF^Neg^: MELAS fibroblast clone harboring negative mutation burden; MF^Hi^: MELAS fibroblast clone harboring high mutation burden.

We further employed a separate coculture system, which allows cell-cell contact between two cell populations while WJMSCs can be removed without intercellular mixture after coculture (Figure 2(a)). This approach created a physical barrier preventing full contact between adjacent cells and thus excluding the formation of intercellular gap junction, while permitting cell-to-cell communication with extruding structures, such as TNTs. To visualize mitochondrial transfer, mitochondria of WJMSCs were labeled with red fluorescence by Cox4-DsRed2 transfection. We did not observe mitochondrial transfer from WJMSCs to MF^Hi^ cells in a separate coculture (Figure S2B). The mutation burden of MF^Hi^ cells was unaffected (Figure S2C). We further labeled mitochondria of WJMSCs and MF^Hi^ cells with red and green fluorescence, respectively, by Cox4-DsRed2 and Su9-EFGP and mixed both cell populations in a blended coculture. No intercellular mitochondrial transfer was observed (Figure S2D). We then pretreated fibroblasts with mitochondrial complex I inhibitor rotenone, by which mitochondrial transfer from donor cells can be induced after recipient cells are prestressed *in vitro* and *in vivo* [22, 27], followed by coculture. Both rotenone-stressed MF^Neg^ and MF^Hi^ cells presented mitochondrial fluorescent signals subsequent to coculture, while MF^Hi^ cells harbored significantly more signal-positive cells than MF^Neg^ cells (Figures 2(c) and 2(d)). The results from the blended coculture were consistent with a separate coculture (Figures 2(e) and 2(f)). On the other hand, F-actin depolymerizing agent cytochalasin B- (350 nM) treated WJMSCs 24 h prior to coculture did not conduct mitochondrial transfer (Figures 2(c)–2(f)). This result implicates the pivotal role of TNTs in delivering these organelles. We next determined whether mitochondrial transfer improves the MELAS mutation burden of MF^Hi^ cells. The mutation burden of MF^Hi^ cells cocultured with WJMSCs was dramatically reduced to an undetectable level in the PCR-RFLP assay, identical to those of WJMSCs and MF^Neg^ cells (Figures 3(a) and 3(b)). Of note, the rescue effect persisted in continuous culture for up to 28 days after coculture (d35) (Figures 3(a) and 3(b)), indicating that improvement of mutation burden is capable of long-term retention. The elimination of mutation burden implies that mtDNA molecules of WJMSCs enter MELAS fibroblasts through mitochondrial transfer. This was confirmed by sequencing hyper variant region 2 (HVR2) of D-loop of the rescued MF^Hi^+WJMSCs. As shown in Figure 3(c), the HVR2 sequence of MF^Hi^ cells cocultured with WJMSCs is identical to WJMSCs, validating the mitochondrial transfer from WJMSCs.

These results suggest that WJMSCs actively transfer mitochondria into stressed MELAS fibroblasts through TNT-dependent communication and transferred normal mtDNA from WJMSCs exhibit long-term retention.

### 3.3. Mitochondrial Transfer of WJMSCs Improves Mitochondrial Translation, Oxidative Stress, and MMP, Remodeling Mitochondrial Morphology and Preserving Bioenergetics of MELAS Fibroblasts

**Figure 4:**
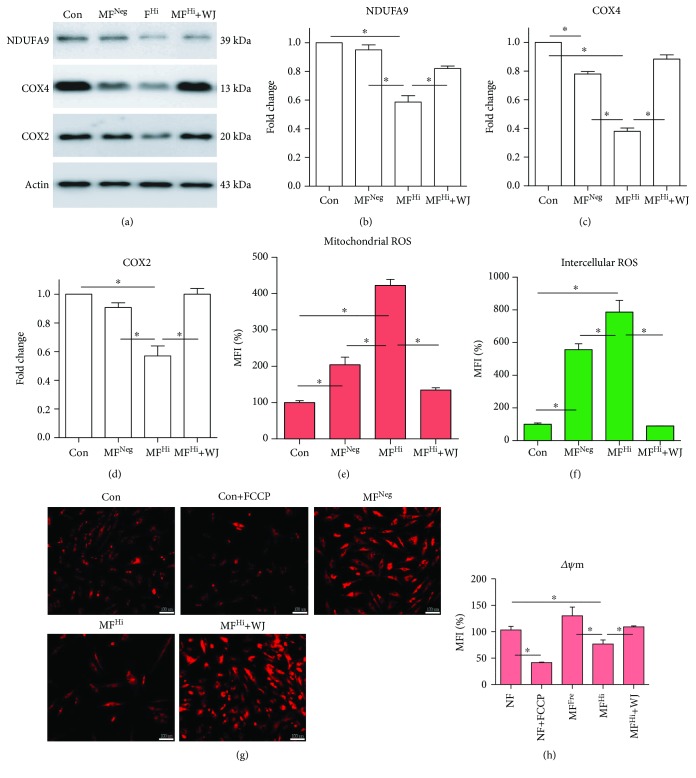
Mitochondrial malfunction of MELAS fibroblasts is improved following mitochondrial transfer. (a) Representative immunoblot image of mitochondrial OXPHOS subunits (nuclear-encoded NDUFA9 and COX4 and mtDNA-encoded COX2). Actin as loading control. (b–d) Quantitative results of protein expression level normalized to actin. (e, f) Mitochondrial and intracellular ROS detected with MitoSOX™ Red and H_2_DCFDA, respectively. (g) Mitochondrial membrane potential (ΔΨm) measured by TMRE stain and photographed using a fluorescence microscope. FCCP used to dissipate ΔΨm served as negative control. Scale bar, 100 *μ*m. (h) Mitochondrial membrane potential quantitatively analyzed using flow cytometry. ^∗^ *p* < 0.05 significantly different when compared to the indicated group. WJ: Wharton's jelly mesenchymal stem cell; Con: control fibroblast from normal human; MF^Neg^: MELAS fibroblast clone harboring negative mutation burden; MF^Hi^: MELAS fibroblast clone harboring high mutation burden; MFI: mean fluorescence intensity.

**Figure 5:**
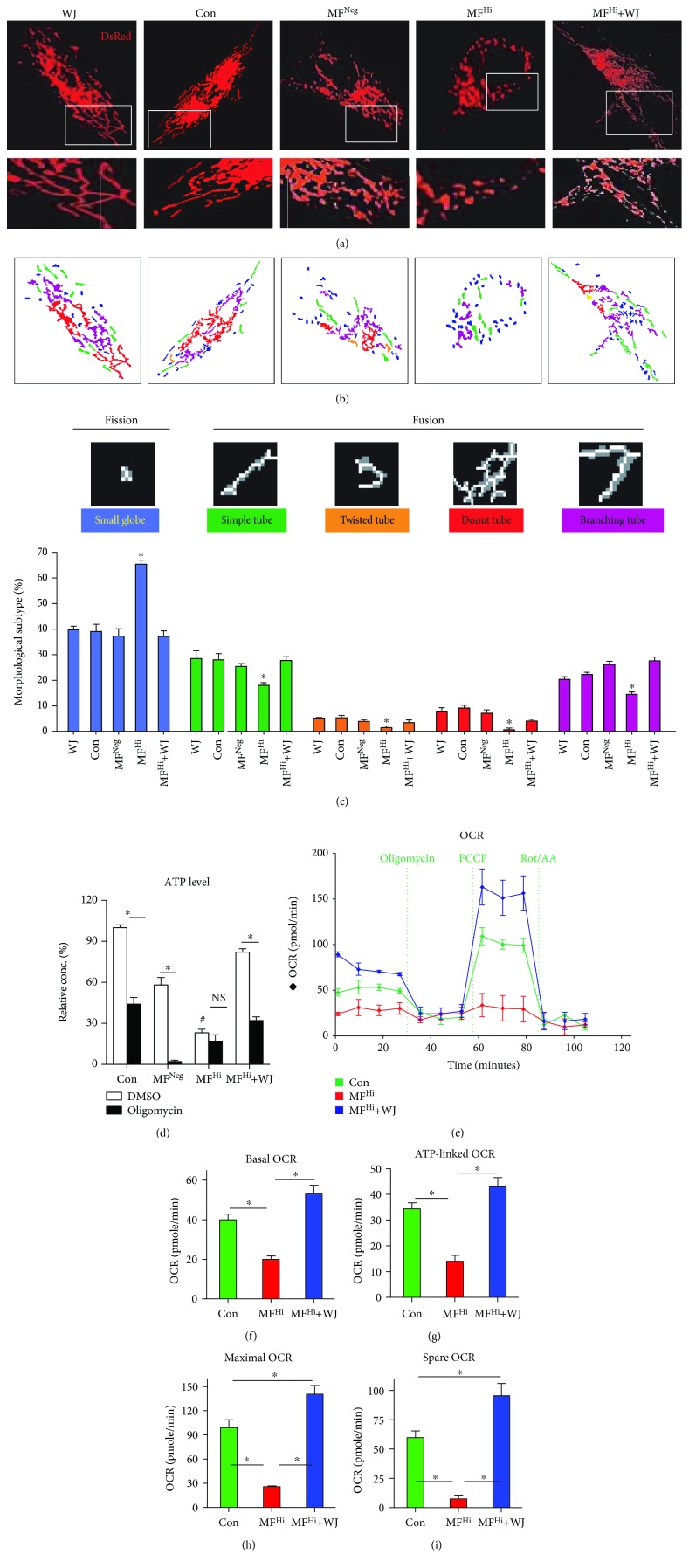
Mitochondrial transfer by WJMSCs contributes to remodel mitochondrial morphology and rescue mitochondrial bioenergetics. (a) Representative image of mitochondrial morphology. Mitochondrial morphology was visualized by transfecting Cox4-DsRed. White rectangle represents highlighted segment enlarged in lower panel. (b, c) The MicroP algorithm categorized mitochondrial morphology into five types: small globe (blue), simple tube (green), twisted tube (orange), donut (red), and branching tube (purple). *N* = 75-400 mitochondria from 15-30 cells and three independent experiments. (c) Quantitative score of mitochondrial morphology. (d) OXPHOS-linked ATP production was dissected with the addition of ATPase inhibitor oligomycin. (e) OCR analysis. Vertical lines indicate the subsequent addition of the ATPase inhibitor oligomycin, the uncoupling reagent FCCP, and the inhibitors of the electron transport chain rotenone/antimycin A (Rot/AA). (f–i) Histograms individually representing basal (basal OCR), oligomycin-sensitive (ATP-linked OCR), FCCP-stimulated (maximal OCR), and the difference basal and maximal respiration (spare OCR). NS: no significance between the indicated groups. ^∗^ *p* < 0.05 significantly different when compared to the indicated group. ^#^ *p* < 0.05 significantly different compared to any other DMSO-treated group. Con: control fibroblast from normal human; MF^Neg^: MELAS fibroblast clone harboring negative mutation burden; MF^Hi^: MELAS fibroblast clone harboring high mutation burden; OCR: oxygen consumption rate; ECAR: extracellular acidification rate.

Following mitochondrial transfer of WJMSCs, we then evaluated mitochondrial function. As RC complex proteins are the central components of OXPHOS, we determined the expression level of nuclear-encoded NDUFA9 and COX4 and mtDNA-encoded COX2. MF^Hi^ cells cocultured with WJMSCs presented increased expression of RC complex proteins (Figures 4(a)–4(d)). As RC complexes play a pivotal role in maintaining ETC and proton gradient across the mitochondrial inner membrane, we then examined ROS production and MMP. Consistent with restoration of RC complexes, ROS production was significantly decreased, while MMP was significantly increased (Figures 4(e)–4(g)). To dissect mitochondrial morphology, cells were transfected with mitochondrial Cox4-DsRed to display mitochondria. MF^Hi^ cells presented markedly fragmented mitochondria, while WJMSCs, control, and MF^Neg^ cells exhibited tubular networks (Figures 5(a)–5(c)). Of note, MF^Hi^ cells cocultured with WJMSCs also presented tubular network morphology (Figures 5(a)–5(c)). We next measured total and OXPHOS-linked ATP production in the absence and presence, respectively, of oligomycin, a mitochondrial ATP synthase inhibitor targeting Fo component of complex V. Control and MF^Neg^ cells showed significantly higher total ATP than MF^Hi^ cells, while the ATP level of MF^Hi^+WJMSCs was significantly improved (Figure 5(d)). Suppression of ATP by oligomycin indicates the energetic dependency on OXPHOS. Compared with oligomycin-sensitive ATP reduction in control and MF^Neg^ cells, MF^Hi^ cells were not responsive, suggesting that MF^Hi^ cells have impaired OXPHOS-linked ATP production (Figure 5(d)). Notably, MF^Hi^ cells cocultured with WJMSCs showed restored OXPHOS-linked ATP production (Figure 5(d)). To further confirm bioenergetics and metabolic phenotype, we measured the oxygen consumption rate (OCR) and extracellular acidification rate (ECAR) using XF24 bioanalyzer. MF^Hi^ cells demonstrated lower basal, ATP-linked, and maximal and spare OCR than control cells, while MF^Hi^ cells cocultured with WJMSCs exhibited significantly improved performance in these assays (Figures 5(e)–5(i)).

### 3.4. Mitochondrial Transfer of WJMSCs Enhances Cell Proliferation, Mitochondrion-Dependent Viability, and Antiapoptotic Resistance to Rotenone

**Figure 6:**
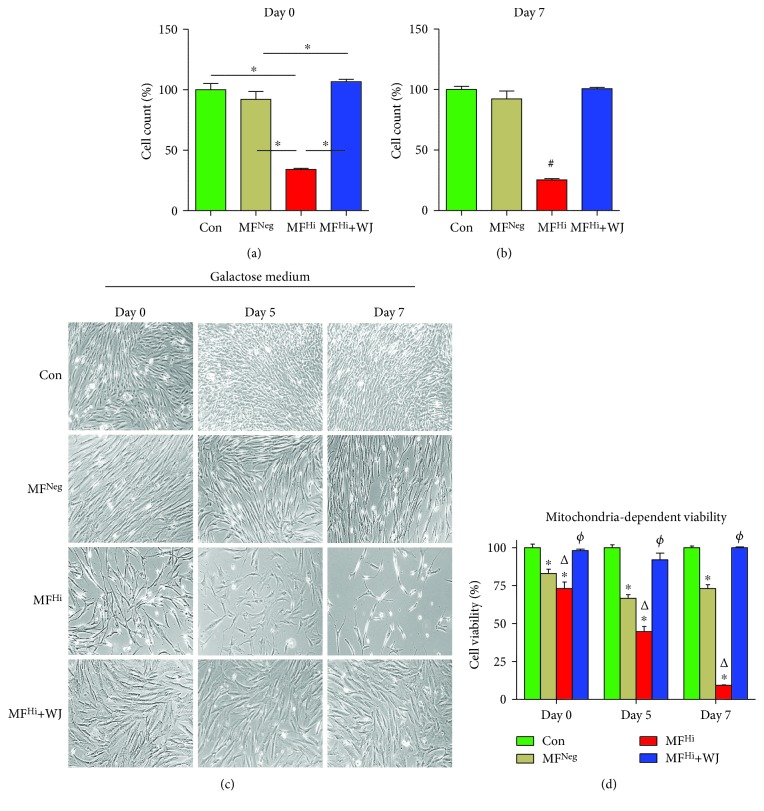
Cell proliferative capacity and mitochondrion-dependent viability are augmented following mitochondrial transfer by WJMSCs. (a, b) 1 × 10^4^ cells were seeded in 12-well plates at the day before day 0. Viable cell number was counted with the trypan blue assay at days 0 and 7. A value expressed as percentage relative to Con. (c) Representative image of mitochondrion-dependent viability. Cells were seeded at the day prior to day 0. Glucose-containing culture medium was replaced with glucose-free 10 mM galactose medium at day 0. Cell confluence photographed and viability detected with CCK-8 kit at days 0, 5, and 7. (d) Quantitative results of cell viability. ^∗^ *p* < 0.05 significantly different when compared to control. ^Δ^ *p* < 0.05 significantly different when compared to MF^Neg^. ^*φ*^ *p* < 0.05 significantly different between MF^Hi^ and MF^Hi^+WJ. Con: control fibroblast from a normal human; MF^Neg^: MELAS fibroblast clone harboring negative mutation burden; MF^Hi^: MELAS fibroblast clone harboring high mutation burden.

**Figure 7:**
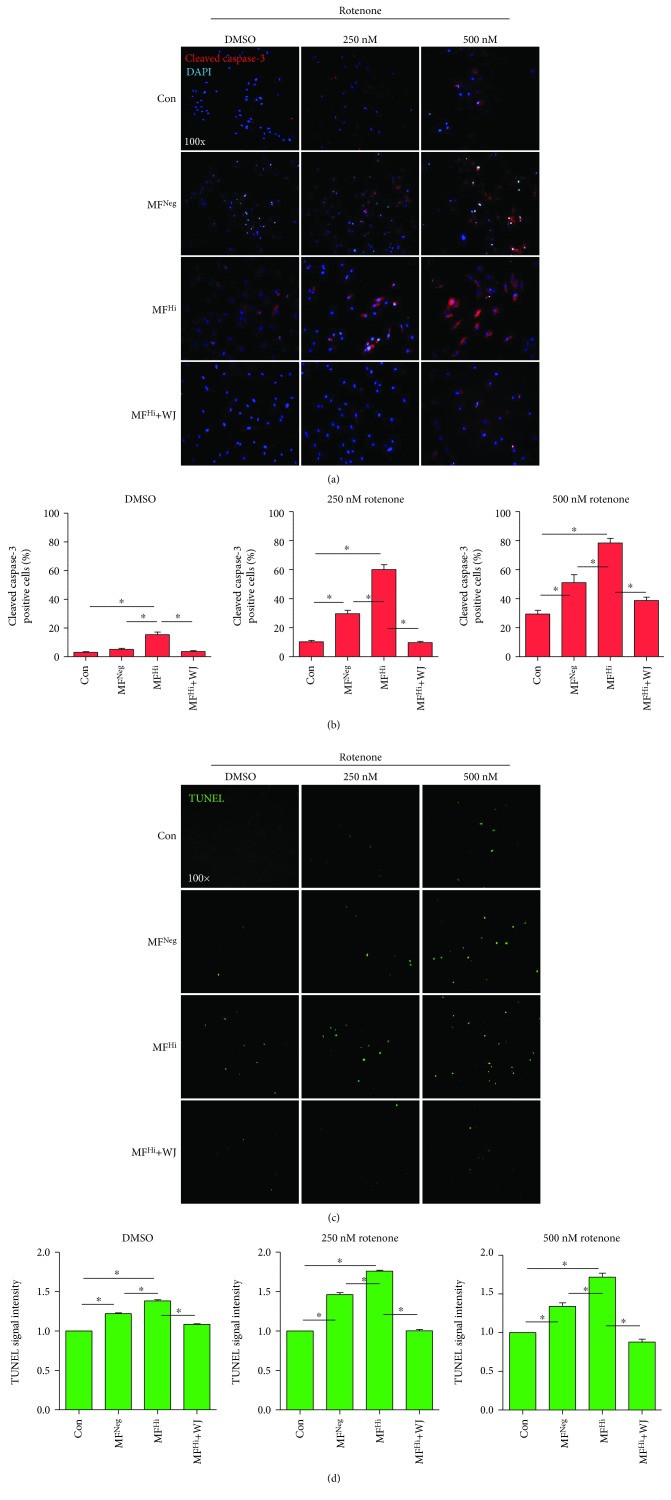
Resistance against apoptotic stress is enhanced following mitochondrial transfer by WJMSCs. (a) Representative image of cleaved caspase-3 stain (red). Nuclear counterstained using DAPI (blue). (b) Quantitative result of positive cleaved caspase-3 relative to DAPI. (c) Representative image of TUNEL stain (green) photographed after cells treated with 0 (DMSO), 250, and 500 nM rotenone for 24 h. (d) Quantitative result of TUNEL-positive signal. ^∗^ *p* < 0.05 significantly different when compared to the indicated group. ^#^ *p* < 0.05 significantly different from every other group. NF: normal human dermal fibroblast; MF^Fre^: MELAS fibroblast clone free of mutation burden; MF^Hi^: MELAS fibroblast clone harboring high mutation burden.

We next examined the cell proliferation rate. MF^Hi^ cells showed a lower cell proliferation rate than control and MF^Neg^ cells, while MF^Hi^ following coculture with WJMSCs had a significantly increased proliferation rate (Figures 6(a) and 6(b)). Additionally, impairment of mitochondrion-dependent viability of MF^Hi^ cells was markedly improved by WJMSCs coculture (Figures 6(c) and 6(d)). This finding is consistent with amelioration of the mitochondrial functions, morphology, and bioenergetics of MF^Hi^ cells (Figures 4 and 5). We then examined cell apoptosis stressed by rotenone by detecting cleaved caspase-3 and TUNEL assay. MF^Hi^ cells presented both a higher cleaved caspase-3 level and TUNEL signal than the control and MF^Neg^ cells under 0, 250, and 500 *μ*M rotenone (Figures 7(a) and 7(b)). Notably, MF^Hi^ cells following coculture with WJMSCs demonstrated significantly reduced expression of both apoptotic indicators (Figures 7(a) and 7(b)), suggesting that mitochondrial transfer from WJMSCs provides apoptotic resistance.

## 4. Discussion

**Figure 8:**
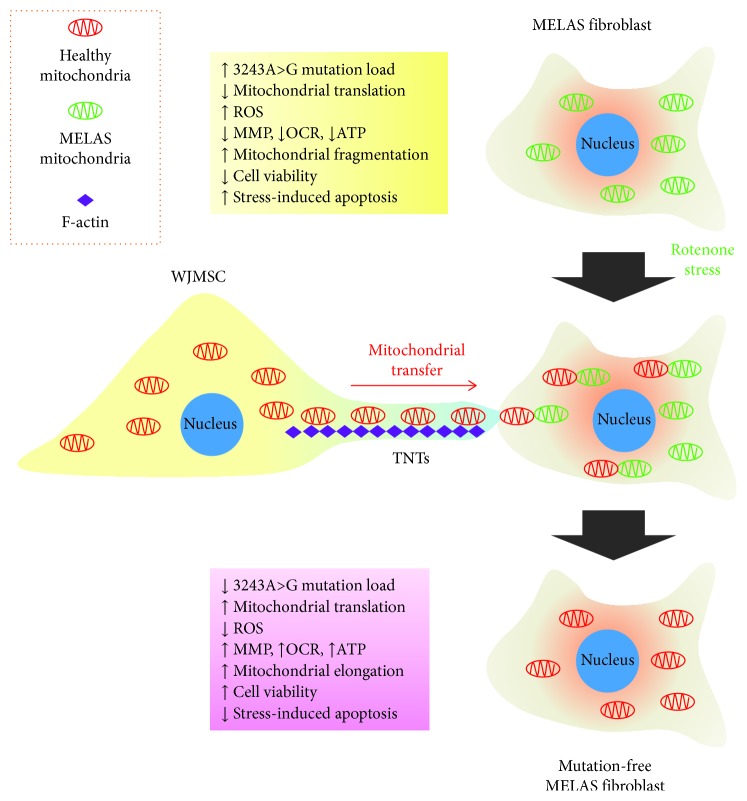
Graphic summary delineating rescue effect of WJMSC-conducted mitochondrial transfer on MELAS fibroblast.

Our team has previously found that umbilical cord-derived WJMSCs yield mitochondrial transfer to mtDNA-devoid *ρ* ^0^ cells and completely restore mtDNA content [21]. We subsequently explored mitochondrial transfer from WJMSCs to cybrid cells harboring the mt.8344A>G mutation from a patient with myoclonic epilepsy with ragged-red fibers (MERRF) [22]. WJMSC-conducted mitochondrial transfer was demonstrated to effectively restore mitochondrial function by replenishing healthy mtDNA and reducing mutation burden. The present study further corroborates these previous observations and extends the investigation into MELAS patient-derived primary fibroblasts, which more closely simulates the physiological state of cells *in vivo*. As illustrated in Figure 8, we demonstrate that human WJMSCs donate healthy mitochondria into rotenone-stressed MELAS fibroblasts and dramatically eliminate mutation burden to a negative level, while the TNT formation inhibitor abolishes WJMSC-conducted mitochondrial transfer, eliminating the mutation burden with proven retention for further 28 days. As well, mitochondrial functions are significantly improved, as indicated by enhancement of protein expression of RC complex components and MMP, as well as decreased intracellular and mitochondrial ROS production. Importantly, mitochondrial bioenergetics, metabolic phenotype, proliferation rate, and mitochondrion-dependent viability of MELAS fibroblasts are significantly improved, to the extent they are comparable with normal fibroblasts. Concurrently with bioenergetics, morphology of mitochondria in MELAS fibroblasts is altered from a fragmented expression to one of tubular elongation. Finally, we observe that antiapoptotic resistance of MELAS fibroblasts is also significantly increased following mitochondrial transfer.

Impaired mitochondrial function of recipient cells may initiate the retrograde signaling triggered by disturbances of ROS, Ca^2+^, AMP/ATP, and the NAD^+^/NADH ratio to activate mitochondrial transfer mechanism of donor cells [28]. Ahmad et al. have shown that MSCs donated more mitochondria to rotenone-treated epithelial cells, which then presented restored mitochondrial respiration and ATP level, as well as decreased mitochondrial ROS production and cytochrome c release [27]. In addition, it has been demonstrated that cellular stresses such as LPS and UV which cause mitochondrial dysfunction of recipient cells are essential for initiation of mitochondrial transfer [15, 29]. Nevertheless, mitochondrial transfer from MSCs to mtDNA-devoid *ρ* ^0^ cells can occur without additional stress [14, 21]. Thus, nearly abolished mitochondrial function may be needed to induce sufficient biological cues necessary for triggering mitochondrial transfer. Accordingly, our study shows that mitochondrial transfer is not successful unless MELAS fibroblasts are stressed by rotenone prior to coculture with WJMSCs (Figure 2 and S2).

Mechanisms accounting for mitochondrial transfer of donor cells include TNT formation, gap junction, and cell fusion [17]. Herein, we observed mitochondrial transfer from WJMSCs to MELAS fibroblasts in a separate coculture system, where contact-dependent gap junction and cell-cell fusion are prevented, and only cellular protruding structures or releasing components are allowed (Figure 2). As such, mitochondrial transfer of WJMSCs is unlikely to be mediated by gap junction or cell fusion. Furthermore, no mitochondrial transfer occurs when WJMSCs are treated with cytochalasin B, which inhibits actin polymerization and tunnel tube formation (Figure 2). This suggests that the formation of TNTs is most likely implicated in mediating mitochondrial transfer of WJMSCs.

The mt.3243A>G mutation, the cause of mitochondrial dysfunction, leads to reduction of mtDNA-encoded proteins, MMP and ATP generation, and increased ROS production [30–32]. Elimination of the mt.3243A>G mutation burden following mitochondrial transfer increases the expression of mtDNA- and nuclear-encoded proteins of mitochondrial RC complexes (Figure 4). As RC complexes are central components of mitochondrial OXPHOS, this fundamental change may contribute to alleviating ROS generation and restoring MMP (Figure 4), ATP production, and metabolic phenotype (Figure 5). As a result of the improved mitochondrial bioenergetics, cell proliferation and mitochondrion-dependent viability of MELAS fibroblasts are significantly improved (Figure 6).

Mitochondrial bioenergetics may change mitochondrial morphology through ATP availability. GTP, formed from ATP by nucleoside diphosphate kinases, serves as a substrate for mitochondrial dynamic proteins, as most of them are GTPases [33]. Increases and decreases of local GTP levels can be sensed by dynamic proteins, such as OPA1 and Drp1, respectively, resulting in mitochondrial fusion and fission [34]. In our study, MELAS fibroblasts having undergone mitochondrial transfer present elongated and fusion morphology, which could be mediated by enhanced bioenergetics (Figure 5). In addition, fission-related morphology facilitates the normal progression of apoptosis, whereas activation of fusion-related proteins antagonizes apoptotic progression [35]. As such, WJMSC-treated MELAS fibroblasts exhibiting resilience against toxin-induced apoptosis (Figure 7) may be attributable to augmented fusion morphology of mitochondria.

Mitochondrial replacement therapy involving replacement of oocyte maternal mtDNA can circumvent mtDNA mutation transmission from a mother to child, thereby preventing generational transmission of mitochondrial diseases [36]. Although mitochondrial replacement therapy is a relatively cutting-edge process, patients suffering from mitochondrial diseases urgently require effective therapy enabling elimination of mtDNA mutation burdens. The finding that bone marrow-derived MSCs (BMMSCs) can donate mitochondria to mtDNA-devoid *ρ* ^0^ pulmonary adenocarcinoma cells initially raised the possibility of treating mitochondrial disorders with stem cells. In terms of cell therapy, BMMSCs which have been primarily exploited in experimental and clinical studies nevertheless possess several drawbacks, including an invasive harvesting procedure and a decreased cellular proliferation and differentiation potential related to donor age and comorbidities [37]. By contrast, umbilical cord-derived WJMSCs are easily accessible, present superior proliferation rate *in vitro*, exhibit less cellular aging, express lower immunogenicity [38], and are devoid of ethical concerns. The FDA has registered dozens of clinical trials on the safety and efficacy of WJMSC transplantation for the treatment of several diseases, such as cardiomyopathies, diabetes, and rheumatoid arthritis. Importantly, clinical studies report promising results with no adverse side effects, apart from several cases of fever [39]. The present study demonstrates the efficacy of WJMSC-based therapy in eliminating mtDNA mutation burdens and restoring the mitochondrial functions of MELAS fibroblasts, presenting a new avenue for treating MELAS and other mitochondrial diseases. The findings herein suggest that the pathophysiological relevance of our results should be further verified in animal model and clinical studies.

## Data Availability

The data used to support the findings of this study are available from the corresponding authors upon request.

## Supplementary Materials

Supplementary Materials Figure S1: (related to Figure 1). Single clones with various mt.3243A>G mutation burdens. Figure S2: (related to Figure 2). Mitochondrial transfer from WJMSCs to MELAS fibroblast is undetectable without pretreatment.
