# Supplementary material for: Mitochondrial Transfer of Wharton's Jelly Mesenchymal Stem Cells Eliminates Mutation Burden and Rescues Mitochondrial Bioenergetics in Rotenone-Stressed MELAS Fibroblasts
Source: Oxid Med Cell Longev. 2019 May 22;2019:9537504. doi: 10.1155/2019/9537504 (PMC6556302; doi:10.1155/2019/9537504)
Supplement: Supplementary Materials — Figure S1: (related to Figure 1). Single clones with various mt.3243A>G mutation burdens. Figure S2: (related to Figure 2). Mitochondrial transfer from WJMSCs to MELAS fibroblast is undetectable without pretreatment. [file 9537504.f1.pdf]

Supplementary materials

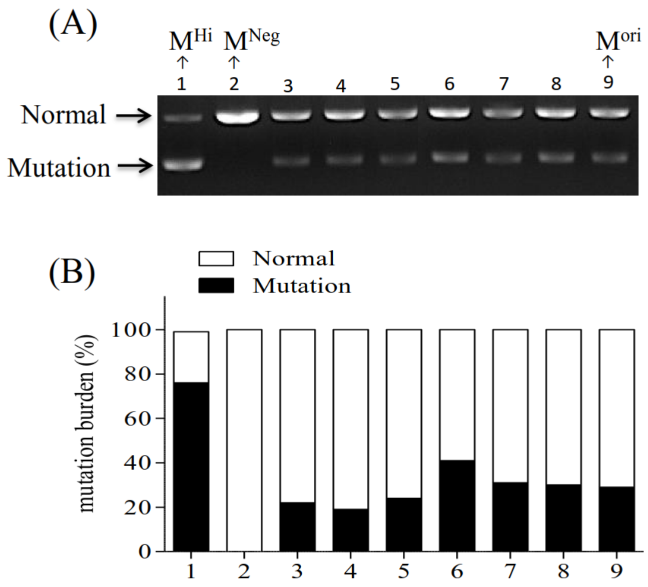

Figure S1 (related to Figure 1). Single clones with various mt.3243A>G mutation burden. Single clones of patient-derived fibroblasts were obtained using clonal dilution. (A) Representative image of mt.3243A>G mutation burden of each fibroblast clone. Mutation (mt3243A>G) was detected using PCR-RFLP. PCR product containing normal mt.3243A was not digested by *Apa I* and showed a 1159 bp band. Mt.3243A>G mutation was *Apa I*-cleaved into 598 and 591 bp band. (B) Quantitative result of mt.3243A>G mutation burden. Con, control fibroblast from normal human. MF^{Ori}, original MELAS fibroblast derived from patient. MF^{Neg}, MELAS fibroblast clone harboring negative mutation burden. MF^{Hi}, MELAS fibroblast clone harboring high mutation burden.

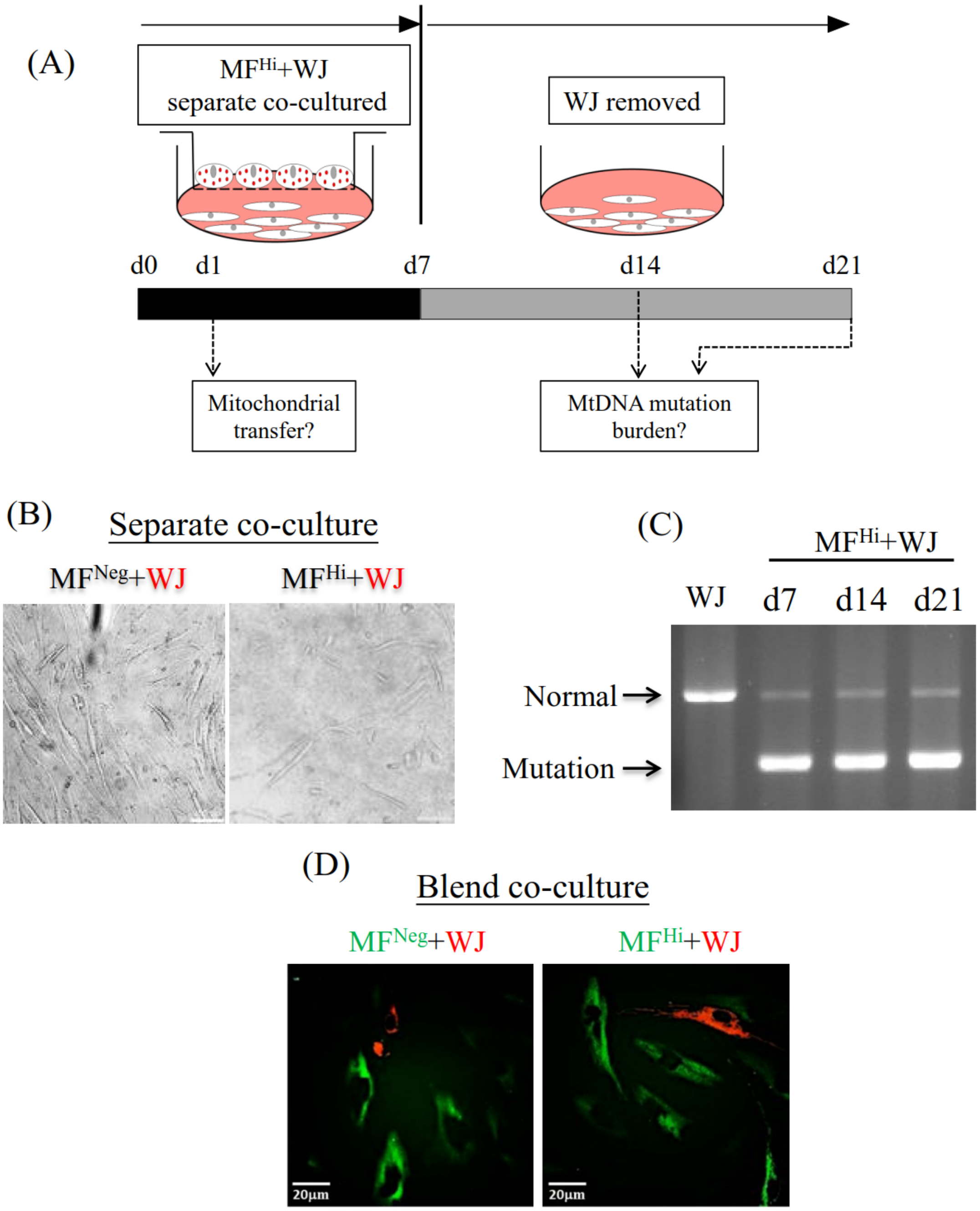

Figure S2 (related to Figure 2). Mitochondrial transfer from WJMSCs to MELAS fibroblast is undetectable without pre-treatment. (A) Schematic of experimental course. (B) Representative image was taken with merging red fluorescence signal with bright field one day after separate co-culture. Scale bar, 100 μm. Note that no mitochondrial transfer was seen. (C) PCR-RFLP showed that mutation burden was unchanged after separate co-culture. (D) Representative image was taken with merging red and green fluorescence signal one day after blend co-culture. Scale bar, 20 μm. WJ, Wharton's jelly mesenchymal stem cell. MF^{Neg}, MELAS fibroblast clone harboring negative mutation burden. MF^{Hi}, MELAS fibroblast clone harboring high mutation burden.
